# Glioblastoma cells induce differential glutamatergic gene expressions in human tumor-associated microglia/macrophages and monocyte-derived macrophages

**DOI:** 10.1080/15384047.2015.1056406

**Published:** 2015-06-05

**Authors:** Judy Choi, Beate Stradmann-Bellinghausen, Eduard Yakubov, Nicolai E Savaskan, Anne Régnier-Vigouroux

**Affiliations:** 1 Johannes Gutenberg University of Mainz; Mainz, Germany; 2 German Cancer Research Center; Heidelberg, Germany; 3 Department of Neurosurgery; Universitätsklinikum Erlangen; University of Erlangen-Nürnberg (FAU); Erlangen, Germany

**Keywords:** glioblastoma, glutamate, monocyte-derived macrophages, tumor-associated microglia/macrophages

## Abstract

Glioblastoma cells produce and release high amounts of glutamate into the extracellular milieu and subsequently can trigger seizure in patients. Tumor-associated microglia/macrophages (TAMs), consisting of both parenchymal microglia and monocytes-derived macrophages (MDMs) recruited from the blood, are known to populate up to 1/3 of the glioblastoma tumor environment and exhibit an alternative, tumor-promoting and supporting phenotype. However, it is unknown how TAMs respond to the excess extracellular glutamate in the glioblastoma microenvironment. We investigated the expressions of genes related to glutamate transport and metabolism in human TAMs freshly isolated from glioblastoma resections. Quantitative real-time PCR analysis showed (i) significant increases in the expressions of *GRIA2* (GluA2 or AMPA receptor 2), *SLC1A2* (EAAT2), *SLC1A3* (EAAT1), (ii) a near-significant decrease in the expression of *SLC7A11* (cystine-glutamate antiporter xCT) and (iii) a remarkable increase in *GLUL* expression (glutamine synthetase) in these cells compared to adult primary human microglia. TAMs co-cultured with glioblastoma cells also exhibited a similar glutamatergic profile as freshly isolated TAMs except for a slight increase in *SLC7A11* expression. We next analyzed these genes expressions in cultured human MDMs derived from peripheral blood monocytes for comparison. In contrast, MDMs co-cultured with glioblastoma cells compared to MDMs co-cultured with normal astrocytes exhibited decreased expressions in the tested genes except for *GLUL*. This is the first study to demonstrate transcriptional changes in glutamatergic signaling of TAMs in a glioblastoma microenvironment, and the findings here suggest that TAMs and MDMs might potentially elicit different cellular responses in the presence of excess extracellular glutamate.

## Abbreviations

GS: glutamine synthetase
HBSS: Hanks' Balance Salts Solution
IL-10: interleukin-10
MACS: magnetic-activated cell sorting
MDMs: monocytes-derived macrophages
MRC1: mannose receptor
NHA: normal human astrocytes
qRT-PCR: quantitative real-time PCR
TAMs: Tumor-associated microglia/macrophages
VEGF: vascular endothelial growth factor

## Introduction

Dysregulation of glutamate signaling in the central nervous system is known to be a key factor in the invasion and growth of glioblastoma^1^ and in induction of seizures.^2^ Glioblastoma cells secrete high levels of glutamate into the extracellular milieu, triggering neuronal cell death through the over-excitation of glutamate receptors and subsequently providing more space for glioblastoma cells to invade and expand.^3,4^

Upon such disruption of brain homeostasis, microglia respond quickly by migrating to the site of disruption, eliciting an inflammatory response, and recruiting peripheral macrophages to the site. Analyses of surgically resected glioblastoma tissues show that as much as 30% of the tissue consisted of tumor-associated microglia/macrophages (TAMs),^5,6^ which are derived from parenchymal microglia in the brain and monocytes-derived macrophages from the blood.^7,8^ These analyses moreover show that the number of TAMs in the tumor region correlated with the histological grade of gliomas.^9,10^ TAMs in the presence of glioma cells become immunosuppressive and elicit an anti-inflammatory response by increasing the expression and release of cytokines such as IL-10 and VEGF, thereby contributing to a milieu that promotes the survival and growth of the glioblastoma cells.^8,11,12^ Microglia express functional glutamate receptors,^13,14^ and microglia as well as macrophages are capable of inducing expression of glutamate transporters and glutamine synthetase in neuropathological conditions,^15^ suggesting a functional role of microglia and macrophages in glutamate signaling. However, little is known about the role of TAMs in response to the dysregulation of glutamate signaling in a glioblastoma microenvironment.

Here we report for the first time altered gene expressions of several glutamatergic signaling markers in TAMs freshly isolated from glioblastoma. To further evaluate possible differences in the response of parenchymal microglia and recruited blood borne macrophages that both constitute the TAMs, we analyzed the gene expressions of the glutamatergic markers in cultured TAMs and monocyte-derived macrophages (MDMs) from healthy volunteers exposed to either glioblastoma cells or normal human astrocytes. The gene expression profile of MDMs co-cultured with glioblastoma cells exhibited a different pattern than that of TAMs. These findings provide new insights on understanding functional differences in glutamate signaling between TAMs and MDMs in glioblastoma microenvironment.

## Results

**Figure 1.**
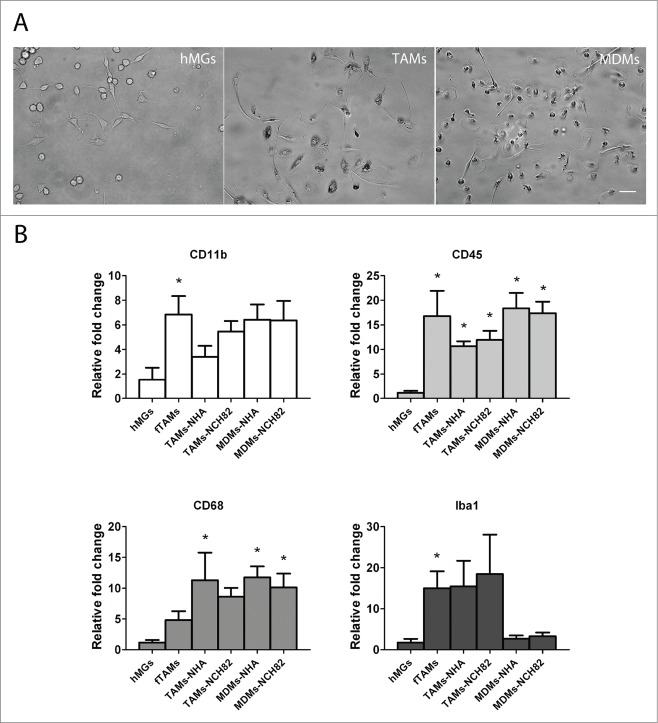
Phenotypic profiles of the various cellular preparations. (**A**) Light microscopy images showing control human microglia (hMGs; left), cultured TAMs (center), and MDMs (right) prior to co-culture exposure. Scale bar = 50 μm. (**B**) Relative quantification of the gene expression levels of microglial/macrophage markers CD11b, CD45, CD68, and Iba-1 from the different cellular preparations and co-culture conditions. Data are given as the mean ± SEM of the relative fold change compared with hMGs of at least 3 independent experiments. * P < 0.05 compared to hMGs.

To investigate the phenotypic profiles of the various cell preparations (microscopy images shown in **Fig. 1A**) used for this study, we analyzed the gene expression levels of 4 microglial/macrophage markers: CD11b, CD45, CD68 and Iba-1 (**Fig. 1B**). Quantitative real-time PCR (qRT-PCR) was performed on the following samples: control adult microglia (hMGs), freshly isolated TAMs (fTAMs), and cultured TAMs or MDMs placed in indirect co-culture (Transwell system) with normal human astrocytes (NHA) or a low-passage human glioblastoma cell line, NCH82, for 48 hours. Control adult microglia, fTAMs, cultured TAMs and MDMs had detectable gene expression levels of all 4 microglial/macrophage markers. Aside from CD68, all microglial/macrophage markers were significantly upregulated in fTAMs compared to hMGs. On the other hand, cultured TAMs and MDMs exhibited different microglial/macrophage profiles when compared to hMGs. Cultured TAMs co-cultured with NHA (TAMs-NHA) had increased gene expressions in CD45, CD68, and Iba-1 (albeit not statistically significant) when compared to hMGs, whereas cultured MDMs co-cultured with NHA (MDMs-NHA) had increased gene expressions in CD11b (also not statistically significant), CD45, and CD68. MDMs had low gene expressions of Iba-1 similar to hMGs. Cultured TAMs or MDMs exposed to NCH82 (TAMs-NCH82 or MDMs-NCH82, respectively) did not alter the gene expression profile of the studied microglial/macrophage markers compared to the respective NHA co-cultures.

**Figure 2.**
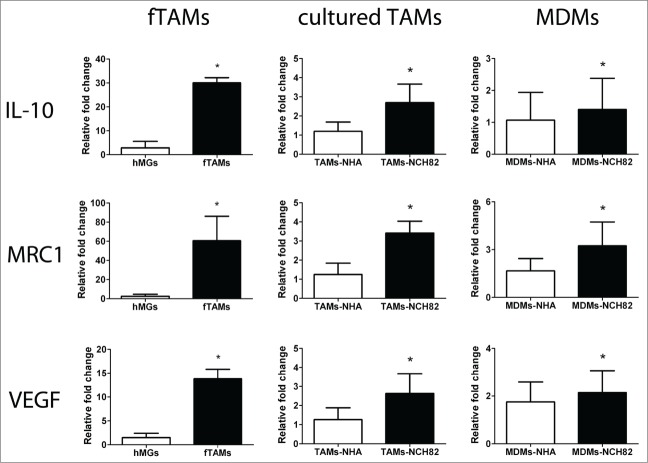
Immunosuppressive profiles of freshly isolated TAMs (fTAMs) as well as cultured TAMs and MDMs after co-culture with NCH82. Relative quantification of the gene expression levels of IL-10, MRC1, and VEGF of fTAMs, TAMs-NCH82, and MDMs-NCH82 showed increase in all 3 genes compared to their respective controls (i.e., hMGs for fTAMs, TAMs-NHA for TAMs-NCH82, and MDMs-NHA for MDMs-NCH82). Data are given as the mean ±SEM of the relative fold change compared with the respective controls of at least 3 independent experiments. * P < 0.05 compared to the respective control of each cellular preparation.

Glioma-infiltrating microglia and macrophages are polarized by the tumor cells toward an anti-inflammatory status.^8^ For example, analysis of TAMs in a murine glioma model has reported the acquisition of the alternative, pro-tumor phenotype of TAMs in the course of tumor development.^11^ Moreover, a study has shown that a 48-hour incubation with glioma cells triggered an alternative, tumor-promoting profile in human monocytes.^16^ Therefore, we studied the alternative, tumor-promoting profile of fTAMs and compared this with cultured TAMs and MDMs after 48-hour indirect co-culture with NCH82 by examining the gene expressions of interleukin (IL)-10, vascular endothelial growth factor (VEGF) and mannose receptor (MRC1), 3 key immunosuppressive molecules expressed in TAMs.^8^ As shown in **Figure 2**, fTAMs exhibited an increased gene expression for the 3 markers compared with hMGs. TAMs-NCH82 showed increased gene expressions of IL-10, VEGF, and MRC1 compared with TAMs-NHA. These increases were also observed to a lesser degree with MDMs-NCH82 compared to MDMs-NHA. This analysis thus confirms an anti-inflammatory status of fTAMs. It further suggests that after maintenance in culture, TAMs still respond to co-culture with glioblastoma cells by eliciting a more immunosuppressive profile than co-culture with normal astrocytes. MDMs co-cultured with glioblastoma cells for 48 hours also elicited the similar immunosuppressive profile.

**Figure 3.**
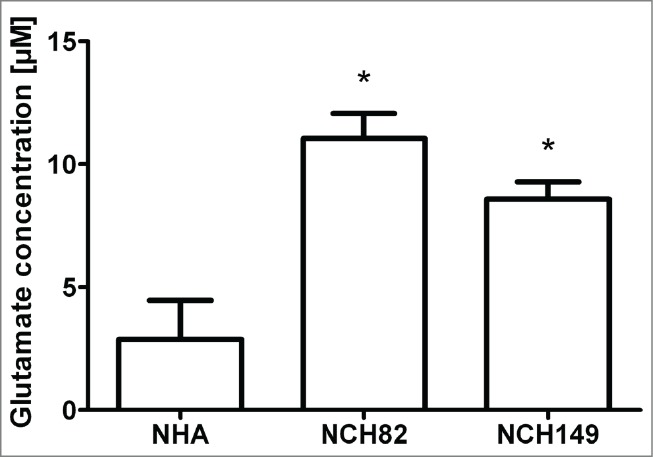
Quantification of the extracellular glutamate level present in the supernatants of NHA, NCH82, or NCH149 cells after 3 d in culture. Both NCH82 and NCH149 cells showed at least a twofold increase of extracellular glutamate level compared to NHA. Data are given as the mean ±SEM of the glutamate concentration expressed in μM of at least 3 independent experiments. *P < 0.05 compared to NHA.

**Figure 4.**
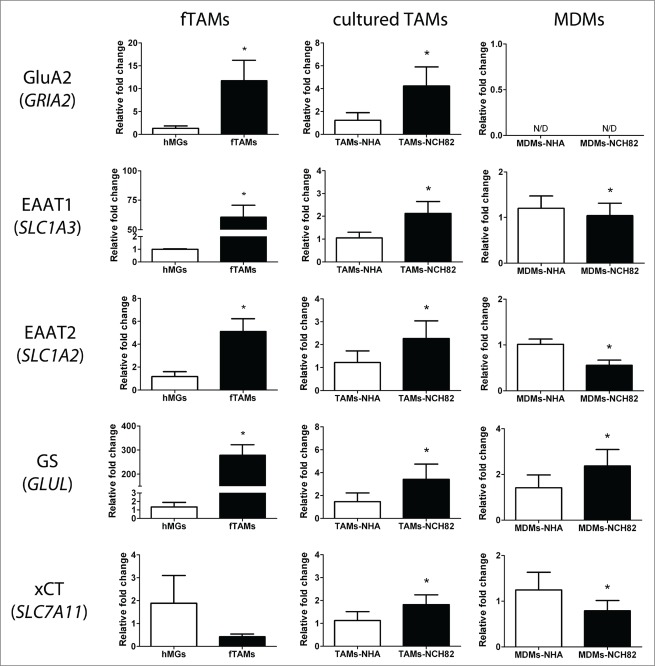
Differential gene expression profiles of glutamatergic genes of freshly isolated TAMs (fTAMs) as well as cultured TAMs and MDMs after co-culture with NCH82. Relative quantification of the gene expression levels of GluA2 (*GRIA2*) showed increased levels in fTAMs and TAMs-NCH82 compared to their respective controls, whereas MDMs showed no detectable levels of GluA2 at either condition. Increased levels of EAAT1 (*SLC1A3*) and EAAT2 (*SLC1A2*) were observed in fTAMs and TAMs-NCH82 compared to their respective controls, whereas MDMs-NCH82 showed decreased gene expression levels of EAAT1 and EAAT2 compared to MDMs-NHA. Increased level of GS (*GLUL*) was observed in fTAMs, TAMs-NCH82, and MDMs-NCH82 compared to their respective controls. Decreased gene expression level of xCT (*SLC7A11*) was observed in fTAMs with near-statistical significance (p = 0.057) and in MDMs-NCH82 compared to their respective controls, whereas TAMs-NCH82 showed slight but significant increase in xCT gene expression compared to TAMs-NHA. Data are given as the mean ± SEM of the relative fold change compared with the respective controls of at least 3 independent experiments. * P < 0.05 compared to the respective control of each cellular preparation.

Glioblastoma cells not only release factors that induce this immunosuppressive profile of TAMs, they are also known to secrete large amount of glutamate in the extracellular milieu. Measurement of glutamate in the supernatants of NCH82 and NCH149 cells after 3 d in culture indicated that these cells release at least more than twofold the amount of glutamate than NHA (**Fig. 3**), proving that these glioblastoma cells release more glutamate than healthy astrocytes. Interestingly, a preliminary microarray analysis of cultured TAMs after a 48-hour incubation with conditioned medium of NCH82 cells showed alterations in several genes relating to glutamate transport and metabolism (data not shown). Therefore, we selected the following 5 of the altered genes: *GRIA2* (also known as GluA2 or AMPA receptor 2), *SLC1A2* (EAAT2), *SLC1A3* (EAAT1), *GLUL* (glutamine synthetase; GS), and *SLC7A11* (the cystine-glutamate antiporter xCT) and determined whether fTAMs, TAMs-NCH82, and MDMs-NCH82 could have alterations in these glutamatergic genes compared to their respective controls. As shown in **Figure 4**, gene expression levels of GluA2, EAAT1, and EAAT2 were significantly increased in fTAMs, whereas the gene expression level of xCT was decreased but with near-statistical significance (p = 0.057). Gene expression of GS was dramatically enhanced almost 300-fold compared to hMGs. TAMs-NCH82 exhibited a very similar profile as fTAMs except for the slight but significant increase in gene expression of xCT.

On the other hand, unlike TAMs, MDMs had no detectable gene expression level of GluA2 in both co-culture conditions, and MDMs-NCH82 showed decreased gene expressions of EAAT1 and EAAT2 compared to MDMs-NHA. The only similarities observed in MDMs-NCH82 when compared to fTAMs were the significant increase in the gene expression of GS and the significant decrease in the gene expression of xCT in MDMs-NCH82 compared to MDMs-NHA. The similar profiles in immunosuppressive markers and glutamatergic markers were also observed in MDMs co-cultured indirectly with another low-passage human glioblastoma cell line, NCH149 (**Fig. S1**). Overall, TAMs and MDMs exposed to glioblastoma cells appeared to exhibit disparate transcriptional responses regarding to glutamate transport.

## Discussion

Glioblastoma cells release high amount of glutamate into the extracellular matrix and are incapable of taking up the extracellular glutamate, resulting in a dysregulation in glutamate signaling.^1^ Under such conditions, astrocytes, the main glutamate scavengers, may no longer suffice to alleviate the neurotoxic levels of extracellular glutamate. The response of TAMs, consisting of both parenchymal microglia and peripheral macrophages recruited to the brain, to the excess of glutamate released by tumor cells is not known. Multiple studies have reported that under neuropathological conditions, microglia and macrophages can induce the expression of glutamate receptors and transporters as well as GS.^17,18,19^ Inflammation is an important component of various neuropathologies and cancer. Pro- and anti-inflammatory molecules have been reported to alter expression of EAAT1, EAAT2 and xCT. A pro-inflammatory milieu appears to lead to increased expression of EAAT and xCT,^19,20,21^ but so do anti-inflammatory molecules.^15,22^ It is worth noting that, in our study, this is an immunosuppressive, anti-inflammatory milieu that led to alterations in gene expression of glutamate receptor and transporters as well as GS that were found to differ between TAMs and MDMs. Furthermore, data from the indirect co-cultures indicate that changes in the glutamatergic genes expression observed in cultured TAMs and MDMs do not depend on direct cell-cell contacts.

Our study featured various cell types and systems: freshly isolated TAMs from resected glioblastoma tissues, isolated human adult microglia, isolated TAMs that were cultured and have been characterized to have regained a less tumor-promoting phenotype,^23^ and monocytes-derived macrophages isolated from healthy volunteers. The observations of increased gene expression levels of CD11b, CD45, and Iba-1 in fTAMs corroborate with previous studies involving TAMs. Human TAMs have been previously observed as CD11b- and CD45-positive,^24^ and it has been reported that CD11b-positive microglia can upregulate CD45 expression in tumor-bearing mice.^25^ Furthermore, Iba-1-positive microglia have been detected to be enriched in the presence of glioma-conditioned medium^26^ as well as in tumor-implanted animals.^27^ Culturing TAMs appeared to change the expressions of some of the microglial markers such as CD45 and CD68 compared to human control microglia, suggesting that the TAMs in culture might still retain some of its phenotypic profile as freshly isolated TAMs. However, similar to fTAMs, co-culture of TAMs and NCH82 did exhibit a more immunosuppressive profile as co-culture of TAMs and NHA, rendering it possible to use this system to investigate the changes in glutamatergic signaling.

As MDMs are bone-marrow derived myeloid cells and are disparate from the yolk sac-derived microglia, it was expected to observe different transcriptional profile in microglial/macrophage markers in MDMs compared to TAMs. In particular, MDMs appeared to have higher CD45 gene expression and lower Iba-1 gene expression than cultured TAMs. On the contrary, gene expression levels of CD45 and CD11b in MDMs appeared to be comparable to the levels observed in freshly isolated TAMs, as described earlier.^24^ These differences in expressions between TAMs and MDMs are worthy of further investigation but are out-of-scope of this study as this study focused on glutamatergic signaling of TAMs and MDMs. Similar to cultured TAMs, MDMs co-cultured with NCH82 also exhibited a more immunosuppressive phenotype than MDMs co-cultured with NHA, therefore allowing further comparison with fTAMs and cultured TAMs.

Increased GluA2 expression in microglia plays an important role in attenuating Ca^2+^ permeability, decreasing its pro-inflammatory response, and subsequently attenuating glutamate-induced neurotoxicity.^28,29^ In addition, glutamate transporters, such as EAAT1 and EAAT2, are known to be responsible for taking extracellular glutamate inside the cells for further conversion into glutamine by GS. Our observation that freshly isolated TAMs and cultured TAMs exposed to glioblastoma cells increase gene expressions of GluA2, EAAT1, EAAT2 and GS suggests that TAMs might compensate for the lack of glutamate uptake and conversion to glutamine by glioblastoma cells and possibly by astrocytes. The increased expression of GS in TAMs should facilitate its conversion into glutamine.^15,30^ Glutamate and glutamine could be partly used by TAMs for their energetic needs, and glutamine could also be released by TAMs to be taken up by glioma cells to fuel up their own energetic and anti-oxidant needs. Glutamate has also been shown to work as a chemoattractant for microglia via AMPA and metabotropic glutamate receptors.^31,32^ Therefore, glutamate released by tumor cells may trigger the GluA2-mediated chemotaxis of resident microglia toward the tumor site.

MDMs, which are immune cells recruited to the tumor from the periphery, elicit a different response in the expression of the glutamate receptor and transporters genes. Interestingly, MDMs alone after co-culture with either astrocytes or glioblastoma cells showed no detectable GluA2 gene expression and showed decreases in EAATs gene expressions after co-culture with glioblastoma cells. It has been previously reported that the MDMs express both EAAT1 and EAAT2 and are capable of taking up extracellular glutamate.^33^ Even though both EAATs in MDMs are decreased in gene expression after glioblastoma exposure, the protein levels might reflect different expression patterns as it had been seen that there were non-similar trends in the gene and protein expressions of EAATs in MDMs after HIV infection.^22^ Therefore, this decrease in gene expression could be a response to the overexpression of EAAT1 and EAAT2 protein in MDMs. This speculation is supported by the observed increase in GS expression by MDMs after exposure to glioblastoma cells, suggesting an increased capacity for conversion of excess levels of intracellular glutamate to glutamine.

The antiporter xCT is responsible for releasing glutamate into the extracellular milieu in exchange of import of extracellular cystine.^34^ While the other tested glutamatergic genes showed increased levels in fTAMs, fTAMs exhibited a near-significant decrease in xCT gene expression compared to hMGs. The decrease in xCT gene expression was observed to be statistically significant in MDMs after co-culture with glioblastoma cells compared to MDMs after co-culture with NHA. A low xCT expression has been reported to be linked to a more anti-inflammatory and neuroprotective phenotype,^15,20,21^ which is a typical phenotype of TAMs. The decreased xCT gene expression might account for the fTAMs and MDMs response to reduce additional release of glutamate into the extracellular matrix.

On the other hand, TAMs in co-culture with glioblastoma cells exhibited a very slight but significant increase in xCT gene expression. It has been reported that increased expressions of both glutamate transporter and glutamate/cystine-antiporter systems cooperatively protected against extracellular glutamate,^35^ providing one plausible explanation for the cultured TAMs response to extracellular glutamate released by glioblastoma cells. The discrepancy in xCT gene expression between fTAMs and cultured TAMs could be due to the culturing conditions and status of activation of cultured TAMs such that cultured TAMs showed higher gene expression of CD68, lower gene expression of CD45 and to a lesser extent of CD11b and had a less immunosuppressive profile than fTAMs.

In presence of large amount of extracellular glutamate, xCT may function as a reverse transporter. In such a case, glutamate uptake by TAMs and MDMs in exchange with cystine being released out of the cell would result in more extracellular cystine available for tumor cells to use, e.g., to synthesize glutathione for their survival and as an anti-oxidant response. Altogether, these data strengthen the therapeutic potential of xCT targeting strategies that would be beneficial to reduce or switch off glutamate metabolism in glioblastoma cells and keep TAMs in a neuroprotective state needed to restore homeostasis after tumor elimination.^36,37^

Collectively, these data suggest a putative neuroprotective role of TAMs by taking up excess extracellular glutamate by increasing expressions of glutamate transporters and glutamine synthetase. This response is not completely mimicked by MDMs exposed to tumor cells, further emphasizing the difference in origin between these bone-marrow derived myeloid cells and the yolk sac-derived microglia. The exact mechanisms of how TAMs or MDMs respond to excess extracellular glutamate in the glioblastoma microenvironment have yet to be elucidated. Protein analyses of the studied genes are necessary to confirm the different responses between TAMs and MDMs, and further studies demonstrating the role of glutamate in eliciting the observed responses, such as uptake and inhibition studies, are warranted.

In summary, this is the first time showing any changes in glutamate signaling in human TAMs and MDMs after exposure to glioblastoma cells. While freshly isolated TAMs as well as cultured TAMs and MDMs after exposure to glioblastoma cells elicit an immunosuppressive response, the transcriptional profiles relating to glutamate signaling of TAMs and MDMs are disparate. Our data strengthens the concept of functional differences between brain microglia/macrophages and monocyte-derived macrophages^38^ and also demonstrates a need to better understand the role of TAMs during dysregulation of glutamate signaling in the glioma microenvironment.

## Materials and Methods

### Isolation and culture of tumor-associated microglia/macrophages (TAMs) from glioblastoma patients

#### Freshly isolated TAMs

Human glioblastoma (Grade IV) tissues (n = 6) were obtained at the University Medical Center Mainz (Germany) in accordance with the local ethical review board and processed within 4–20 h after surgery. Isolation of TAMs was performed using a modified version as described previously.^23^ Briefly, tissues were weighed, mechanically dissociated, and washed in Hanks' Balance Salts Solution (HBSS; Sigma-Aldrich). The tissues were then enzymatically digested with 10 mL/g of tissue of HBSS with Dispase II (1.5 U/mL; Roche) and DNase (250 U/mL; Roche) for 1 h at 37°C and filtrated with a 100-μm and a 40-μm cell strainers (BD Falcon). The pelleted cells were then incubated in erythrocyte lysis buffer^23^ for 10 min on ice. Reaction was stopped with complete Dulbecco's Modified Eagle Medium (cDMEM) consisting of DMEM (Sigma-Aldrich), 10% heat-inactivated fetal calf serum (vol/vol) (FCS; Sigma-Aldrich), 2 mM L-glutamine (Life Technologies), and 50 μg/mL gentamycin (Life Technologies) and centrifuged. CD11b^+^ TAMs were isolated by magnetic-activated cell sorting (MACS) with the CD11b isolation kit (Miltenyi Biotec) according to the manufacturer's instructions. Sorted cells were pelleted and frozen for later RNA isolation.

#### Cultured TAMs

Human glioblastoma tissues (n = 4) were obtained at the Department of Neurosurgery at University Heidelberg Hospital (Heidelberg, Germany) in accordance with the local ethical review board and processed within 4–20 h after surgery. TAMs were isolated as described above except that the MACS sorting step was replaced by a differential adhesion step. The detailed isolation protocol and characterization of the cultured TAMs were previously described in details.^23^ After isolation, the TAMs were kept in culture in cDMEM supplemented with 10 ng/mL M-CSF (R&D Systems) for one week and subsequently cultured in cDMEM without M-CSF for another week before co-culture experiments. **Figure 1A** shows a light microscopy image of 2-week old cultured TAMs prior to co-culture exposure.

### Isolation of primary human adult microglia

Surgical specimens from epileptic patients were obtained from the Neurosurgical staff at University Hospital Erlangen (Erlangen, Germany) in accordance with the local ethical review board and processed within 2 h after surgery. Isolation of microglia was performed according to published protocols^39^ with some modifications. The specimens were carefully stripped of meninges and vessels, minced, enzymatically digested with 10 mL/g of tissue of HBSS (Life Technologies) with papain (2.5 U/mL; Worthington) and DNase (10 U/mL) for 1 h at 37°C and filtrated with a 100-μm cell strainer and dispersed into a single-cell level. Cells were maintained in DMEM containing 4.5 g/L glucose, L-glutamine, pyruvate, 10% FCS (Biochrom). Medium was replaced every 4 d. Microglia were isolated by collecting the floating fraction of mixed glial cultures following 90 min on a rotary shaker at 37°C at 250 rpm. Attached microglia were allowed to recover for 24 h prior to RNA extraction. **Figure 1A** shows a light microscopy image of microglia prior RNA isolation.

### Isolation and culture of human monocyte-derived macrophages (MDMs)

Human peripheral blood mononuclear cells (PBMCs) were obtained from buffy coats of healthy donors (n = 6) from the blood bank of the Heidelberg University Hospital. PBMCs were isolated by Ficoll Paque (GE Healthcare) density gradient centrifugation. CD14^+^ monocytes were then extracted from freshly isolated PBMCs by MACS with the CD14 isolation kit (Miltenyi Biotec) according to the manufacturer's instructions. The purity of the extraction was >98% of CD14+ monocytes as inspected by flow cytometry (data not shown). Isolated monocytes were differentiated into MDMs by culturing them in RPMI1640 medium (Sigma-Aldrich) with 10% FCS, 2 mM L-glutamine, 1% penicillin-streptomycin (Life Technologies), and 10 ng/mL M-CSF for 12–14 d. **Figure 1A** shows a light microscopy image of MDMs prior to co-culture exposure.

### Cell culture of glioblastoma cell lines and normal human astrocytes

Human primary glioblastoma cell lines (NCH82 and NCH149^40^) were grown in cDMEM. Normal human astrocytes (NHA; ScienCell) were cultured in complete astrocyte medium (ScienCell), prepared according to the manufacturer's instructions.

### Co-culture setup of TAMs or MDMs with glioblastoma cell lines or normal human astrocytes

A total of 2 × 10^5^ TAMs or MDMs were seeded in cDMEM for each well of a 6-well plate. A total of 10^5^ NCH82 or NCH149 glioblastoma cells (only with MDMs) or 2 × 10^5^ NHA were seeded in cDMEM onto Transwell inserts with 0.4-μm membrane (BD Falcon). Cells were plated in culture for 48 hours before harvesting.

### Glutamate quantification

Glutamate concentration in the supernatants of NHA, NCH82, and NCH149 (10^5^ NCH82 or NCH149 or 2 × 10^5^ NHA seeded in a well of a 6-well plate) was measured after 2 d in culture with cDMEM using the Glutamine/Glutamate Determination Kit (GLN-1, Sigma-Aldrich) according to the manufacturer's instructions. Absorbance was read at 340 nm using a Tecan Infinite M200 plate reader (Tecan).

### RNA isolation and quantitative real-time PCR

Total RNA were isolated from control human microglia, fTAMs, cultured TAMs, and MDMs using RNeasy Mini Kit (Qiagen) according to the manufacturer's instructions. The quality of RNA was tested using the 2100 Bioanalyzer (Agilent Technologies). Reverse transcription was performed using the Omniscript RT Kit (Qiagen) according to the manufacturer's instructions. Quantitative real-time PCR was performed using the ABI 7300 or the StepOnePlus real-time PCR instruments (Life Technologies). Each reaction consisted of cDNA samples, Taqman Universal PCR master mix (Life Technologies), and human Taqman primer/probe sets (Life Technologies). The primer/probe sets used in this study are provided in **Table 1**. Relative fold changes in gene expression were determined using the 2^−ΔΔCt^ method.^41^

**Table 1.** Taqman gene expression assays used for quantitative real-time PCR of the studied genes

Gene name	Protein name	Assay ID
18S*		Hs99999901_s1
*ITGAM*	CD11b	Hs00355885_m1
*PTPRC*	CD45	Hs04189704_m1
*CD68*	CD68	Hs02836816_g1
*AIF1*	Iba-1	Hs00610419_g1
*IL10*	IL-10	Hs00961622_m1
*MRC1*	Mannose Receptor	Hs00267207_m1
*VEGFA*	VEGF-A	Hs00900055_m1
*GRIA2*	GluA2	Hs00181331_m1
*SLC1A3*	EAAT1	Hs00188193_m1
*SLC1A2*	EAAT2	Hs01102423_m1
*GLUL*	GS	Hs01013056_g1
*SLC7A11*	xCT	Hs00921938_m1

* internal reference gene.

### Statistical analysis

Values are expressed as mean ± SEM. Each group consisted of at least 3 independent trials for each condition studied. The statistical significance of the differences was determined using the student's t-test (for 2 groups) or one-way ANOVA (for more than 2 groups) followed by Student-Newman-Keuls post-hoc analysis. Significance was set at p < 0.05.
