# Supplementary material for: Glioblastoma cells induce differential glutamatergic gene expressions in human tumor-associated microglia/macrophages and monocyte-derived macrophages
Source: Cancer Biol Ther. 2015 Jun 5;16(8):1205–13. doi: 10.1080/15384047.2015.1056406 (PMC4623498; doi:10.1080/15384047.2015.1056406)
Supplement: Supplemental Figure 1 [file kcbt-16-08-1056406-s001.docx]

**

Supplementary Figure 1. Genomic profiles in immunosuppression and glutamatergic signaling of cultured MDMs after indirect co-culture with another low-passage human glioblastoma cell line, NCH149.** (A) Relative quantification of the gene expression levels of IL-10, MRC1, and VEGF of MDMs-NCH149 showed increase of all 3 genes compared to MDMs-NHA. (B) Relative quantification of the expression levels of *GRIA2*, *SLC1A3*, *SLC1A2*, *GLUL*, *SLC7A11* in MDMs-NCH149 showed similar expression pattern as MDMs-NCH82 when compared to MDMs-NHA. Data are given as the mean ± SEM of the relative fold change compared to MDMs-NHA of at least three independent experiments. * P < 0.05 compared to MDMs-NHA.
